# Potential Benefits and Drawbacks of Virtual Clinics in General Surgery: Pilot Cross-Sectional Questionnaire Study

**DOI:** 10.2196/12491

**Published:** 2020-01-13

**Authors:** Emily Rutherford, Roghinio Noray, Caolán Ó hEarráin, Kevin Quinlan, Aisling Hegarty, Lenin Ekpotu, Chinedum Arize, Fiyinfoluwa Fabamwo, Abdulaziz Alrubaiaan, Avinash Bhupalan, Abdulla Alshehhi, Colm Power, Arnold David Konrad Hill

**Affiliations:** 1 Department of Surgery Royal College of Surgeons in Ireland Dublin Ireland

**Keywords:** telemedicine, surgery, outpatient care, remote consultation, delivery of health care

## Abstract

**Background:**

Escalating demand for specialist health care puts considerable demand on hospital services. Technology offers a means by which health care providers may increase the efficiency of health care delivery.

**Objective:**

The aim of this study was to conduct a pilot study of the feasibility, benefits, and drawbacks of a virtual clinic (VC) in the general surgical service of a busy tertiary center.

**Methods:**

Patient satisfaction with current care and attitudes to VC were surveyed prospectively in the general surgical outpatient department (OPD; n=223). A subset of patients who had undergone endoscopy and day surgery were recruited to follow-up in a VC and subsequently surveyed with regard to their satisfaction (20/243). Other outcomes measured included a comparison of consultation times in traditional and virtual outpatient settings and financial cost to both patients and the institution.

**Results:**

Almost half of the patients reported barriers to prospective use of VCs. However, within the cohort who had been followed-up in the VC, satisfaction was higher than the traditional OPD (100% as compared with 187/223, 83.9%). Significant savings in both time (*P*=.003) and financial costs to patients and the institution were found.

**Conclusions:**

For an appropriately selected group of patients, VCs offer a viable alternative to traditional OPD. This alternative can improve both patient satisfaction and efficiency of patient care.

## Introduction

### Background

As the global population continues to grow, pressure on health care systems is increasingly evident. This is apparent in developed countries, where an increasing proportion of gross domestic product is spent on health care costs [1], and also in the developing world as noncommunicable disease costs escalate [2]. Just as financial costs associated with health care provision are on the rise, so is the time commitment by physicians looking after increasingly complex patients with multiple comorbidities [3]. To address these issues, technological advances are one possible component of a solution to the challenges faced by health care systems worldwide. Although the use of technology is only one part of a larger policy response to ongoing health care provision issues, it represents an area in which significant improvement of services may be made. Virtual clinics (VCs) are at the forefront of this technological health care innovation.

Virtual consultation is a broad term that describes a form of nonphysical contact (eg, over the telephone, a video link, or other Web-based platform) between a patient and their health care provider(s), or between 2 or more health care providers, to encourage collaborative efforts between physicians to ensure the best possible outcome for the patient. There are different types of virtual consultations, examples of which may include the following: (1) Patient-general practitioner (GP), in which the patient calls the GP on telephone or video call as opposed to physically going to the clinic [4]; (2) GP-consultant, in which the GP contacts a specialist doctor regarding the joint management of a patient between the community and a specialist center [5]; (3) GP-multidisciplinary team (MDT), whereby GPs call into MDT meetings to learn from experts and acquire clinical skills [6] as well as to receive assistance with the management of less severe cases, thereby allowing health care resources to be reallocated to patients with more serious or complex cases [7]; and (4) Patient-specialist, in which the patient contacts a specialist doctor to receive care for a specific condition [8].

The potential advantages of VCs are evident and include reduced waiting times [9], decreased travel times to and from health centers [10,11], increased utilization of specialist knowledge [12], and increased efficiency of appointments and streamlining of referrals [13]. However, valid concerns exist regarding the safety of patient data, acceptability of this model to patients and clinicians, and feasibility of implementation [14]. Although a compelling argument for increased efficiency and cost-saving measures does exist, this must be balanced against patient safety and acceptability and developed with due regard to integration into current services.

Overall, the advantages of VCs may include higher patient satisfaction [5], more time-efficient appointments, reduced travel costs [10] and waiting times in outpatient department (OPD), and increased efficiency in the use of health care resources [15]. However, on the other hand, it has brought about reasonable concerns with regard to practicality [16], data breaches, patient privacy, and confidentiality [17], technical challenges, as well as some apprehension regarding the lack of face-to-face interaction and physical examination [18]. Other disadvantages such as limited capability, differing internet access, and concerns among both patients and the medical community remain. It is clear that blanket application of *one size fits all* VCs is inappropriate and that any integration into current systems must follow a structured and evidence-based approach [19]. It is also clear that there are issues and attitudes that still must be addressed before VCs can become a part of worldwide health care. For a comprehensive overview of the development of the field, including a conceptual overview and discussion of barriers to use, the reader is directed to reviews by international groups [16,20,21]. However, as technology develops and specific populations are considered, ongoing appraisal of the role of technology in health care is crucial. Thus, an investigation into the suitability of VCs in specific clinical areas is an important area of study.

### Objectives

In 2016, Beaumont Hospital Dublin launched a pilot VC project in partnership with an Irish telemedicine provider, VideoDoc [22]. This platform was initially conceived as a substitute for primary care, in which a video link between a patient and GP could be used to conduct a consultation. Additional facilities for prescribing and documentation were included in the technology. An expansion of this platform into the hospital system, therefore, trialed video consultation instead of OPD follow-up for selected general surgical patients. Inclusion criteria included follow-up for operations such as laparoscopic cholecystectomy, hernia repair, or appendectomy as well as the ability to use the necessary technology. Similarly, patients with benign biopsy results (breast and thyroid) and endoscopy (+/− needing additional surveillance) were consulted over the telephone, instead of traveling to the tertiary center and waiting for a long time in the OPD to receive benign results. The aim of the pilot project was to assess the feasibility of the use of VCs in the surgical service. Outcomes such as time efficiency of VCs as compared with standard outpatients; economic considerations; and patient attitudes, both prospectively and retrospectively, were also examined.

## Methods

The objective of this pilot study was to assess the potential benefits and drawbacks of a VC system embedded within a larger general surgical population. Metrics included the following: (1) prospective patient attitudes to the concept of a VC; (2) retrospective attitudes in a smaller cohort; (3) a description of efficiency of standard outpatient care as compared with VCs in terms of waiting times and consultation times; and (4) a preliminary estimation of economic benefit of VCs to patients and the institution, without formal in-depth analysis of the economic impact of policy.

To assess the attitude of patients toward VCs, a survey was drafted with 17 questions (Multimedia Appendix 1). Questions 1 to 13 related to the current outpatients setting, asking for details regarding travel (1-7) and patient satisfaction (8-13). Patient satisfaction was assessed with the use of ordinal questions, ie, answers were given on a Likert scale from 1 (strongly disagree) to 7 (strongly agree). Questions 15 to 17 related to the views of patients in relation to the concept of VCs. Answers were organized in a dichotomous format (though question 17 had a space for patients to give a reason if they would not want to attend a VC). Surveys were distributed twice a week in the outpatient clinic of 2 general surgical consultants with the clinicians’ permission, under registered audit CA340 in Beaumont Hospital from January 2018 to April 2018. An announcement was made at the outpatient reception by a clinical staff member regarding the survey; thereafter, patients who wished to partake indicated their willingness to do so. Both a morning and afternoon clinic were utilized to generalize findings.

Patients who had used the VC during the pilot study were also presented with a modified version of the survey to assess their experience and identify any problems and make improvements where necessary (n=20). A total of 20 patients who had undergone care in the VC were selected at random. The 20 patients were “seen” in the VC for postprocedure follow-up, eg, instead of follow-up in the OPD. These patients had been recruited to the VC follow-up at the time of their discharge from hospital. A protocol was employed whereby patients were contacted at 3 separate time points only before cessation of contact attempts to minimize patient burden. Overall, between prospective and retrospective cohorts, 243 participants completed the surveys.

In addition to the analysis of patient attitudes and satisfaction, we sought to demonstrate the efficiency of a VC by comparing the time taken for a senior house officer to see 10 patients in the VC against the time taken to see the same number of patients in the OPD. Only OPD patients who fit the previously mentioned VC inclusion criteria had their consultation times recorded. The time taken for a patient to be seen in the VC was provided to us by the VideoDoc app itself. In addition to this information, we were also provided with patients’ waiting times between logging on to the app and being seen by a doctor as well as their satisfaction with the VideoDoc experience.

A comparison of average costs between the VCs and traditional clinics was also compiled using information provided by the hospitals’ department of finance. In this manner, both patient and provider costs were assessed.

## Results

### Travel and Waiting Times

The data collected during this project were obtained from the patients of 2 general surgical consultants at a busy tertiary center in Dublin, Ireland, over a period of 15 weeks, using a survey handed out to a total of 223 patients. Separately, a subset (n=20) of patients who had used the VC in Beaumont were surveyed after their appointment to ascertain patient satisfaction and evaluate the new virtual service.

The first component of the survey looked at the travel requirements for attending an outpatient appointment.

The average one-way travel time from the patients’ respective homes to Beaumont Hospital for their appointment was found to be 43 min (range: 2-180 min; median: 30 min; SE 2.44; SD 35). The average time spent waiting to be seen by a clinician was 61 min (range: 3-240 min; median: 60 min; SE 3.16; SD 41), underlining the fact that the patients spend more time waiting to be seen than they do commuting to the hospital

The median cost incurred by the patients during their commute to the hospital was calculated at a value of €10 (range: €0-100; mean €12.50; SE 1.13). The average number of work days missed to attend the outpatient appointment was 0.85 days, with varying levels of lost earnings for this time

### Patient Attitudes Toward the Virtual Clinic

Another survey component dealt with patients’ opinion on the use and application of VCs and whether or not they would be open to this model of care. This showed that 52.0% (116/223) of patients believe that the physician can still provide care without being able to perform a physical examination at every appointment.

Importantly, 88.8% (198/223) of patients are of the view that physical examination is an important part of a consultation. When asked whether they would attend a VC over an outpatient appointment, data showed that 48.9% (109/223) said no, with 43.0% (96/223) saying they would. If they did attend a VC, however, 57.8% (129/223) reported no issue with answering personal questions. When asked to take into account the time and cost it takes to come to an outpatient appointment and compare it with that of a VC, which would they prefer to attend, 55.2% (123/223) of patients prefer OPD despite the downsides, 30.9% (69/223) chose VC, and 4.0% (9/223) had no preference.

### Patient Attitudes Toward the Current Model of Care

Finally, another section of the survey is the patient satisfaction component, as it relates to the current “traditional” OPD. A total of 83.0% (185/223) of patients strongly believed that taking an active role in their own health care is important. Moreover, 87.9% (196/223) of patients were pleased with the quality of the medical appointment. In addition, 74.0% (165/223) of patients agreed that their appointment was on time and efficient. Patients found the appointments to be conducted in a confidential manner, with 81.2% (181/223) strongly agreeing and 87.0% (194/223) in total agreeing to this point. Patients had no problems disclosing personal information, as 88.9% (198/223) of patients felt comfortable sharing personal information with their health care provider. Overall, 83.0% (185/223) of patients were satisfied with their appointments within the current framework.

### Retrospective Patient Attitudes Toward the Virtual Clinic

A separate cohort of patients who had attended the VC were selected at random and surveyed with a modified version of the questionnaire to assess their satisfaction level and their opinion on the outcome of their health care (n=20). A total of 100% of the patients found the technical quality to be acceptable and the appointment to be very time- and cost-effective and conducted in a confidential manner. All of this cohort believed that the outcome of their care was exactly the same as if they were to attend an outpatient appointment and meet their doctor in person, and they were overall satisfied with the appointment.

### Financial Impact of New Technologies on Hospital

The stated cost per patient was, on average, €158.92 per general surgery patient in the OPD, resulting in an annual cost of €553,995 (Beaumont Finance Department). When the salary of both administrative and clinical staff was taken into account, it costs the hospital an average of €14 to see a patient in the VC, as based on the average time of less than 10 min to see a patient and complete the associated documentation (n=10). However, this was predicated on free usage of the technological platform as sponsored by the private company.

The length of the average consultation in VideoDoc was 5 min and 19 seconds (range: 2-14 min; SD 4.1), with an average waiting time of 4 min and 40 seconds. This was skewed somewhat by 1 user who had some technical difficulties and needed assistance using the app. Overall, a representative sample of patients had a total waiting and consultation time of less than 10 min. The length of the average OPD consultation for similar matched patients was 14 min (range: 3-24; SD 6.7). An unpaired 2-tailed *t* test assuming unequal variances showed that these were statistically significantly different (*P*=.003).

## Discussion

### Principal Findings

The OPD sees more than 143,000 patients per year [7]. However, up to 15% of patients miss their appointments [23], and there are many patients who have to wait for an extended period for an appointment because of minimal availability. The Irish Times reported that there were 478,569 people waiting for OPD appointments as of May 2017 [24,25]. Our research has shown that it takes a patient approximately 43 min on average to travel to Beaumont Hospital, after which they are checked in at the reception and have to wait for an even longer period, estimated at 61 min, before they are called in by the doctor. Therefore, it takes patients a total of 104 min to attend an outpatient appointment, which includes both one-way travel time to the hospital and waiting time at the reception. This means that for every 1 patient waiting to be seen in the OPD, approximately 5 patients can be seen by VC, given that our data show that VC only takes 10 min, even allowing for note-taking and administrative tasks in between consultations.

### Potential Financial Impacts

It is important to note that time off work has to be taken to attend these appointments; on average, patients had to take a full day off, with the majority of this leave of absence being unpaid. Some patients also had to be accompanied by a relative or friend. On the other hand, a VC appointment is not associated with any travel time and very little waiting time as the doctor and patient are both available at the scheduled time of appointment. The clinic in the pilot project ran outside typical work hours between 5 to 6 pm to allow patients to attend a full day of work and conveniently attend their appointment after working hours, without missing a day’s pay.

Furthermore, in this pilot program, use of the VC was free of charge for the selected VC patients and, thus, was associated with no additional travel cost. Recalling that the average travel cost for an outpatient appointment was €10 on top of lost wages/ productivity, our data suggest from a patient perspective that VCs are certainly the more cost-effective option. Access to the requisite technology is an important consideration in an equitable health care system that incorporates a virtual aspect; however, 97% of the adult Irish population have access to a mobile phone [26]. Furthermore, internet access in urban areas in Ireland is generally of high quality, with a national plan in place to improve rural broadband coverage over the next 7 years [27].

In addition, the use of the VC saves the hospital and the health care system a considerable amount. It costs the hospital an average of €158.92 per general surgery patient in the OPD, resulting in an annual cost of €553,995 (Beaumont Finance Department). When the salary of both administrative and clinical staff was taken into account, it costs the hospital an average of €14 to see a patient in the VC.

This leads us to conclude that if VC were to become the mainstay of follow-up care, there would be an increase in the total number of patients seen on a daily basis and a decrease in the number of missed appointments. Although VC is not suitable for everyone (as video clinic is clearly inappropriate for very elderly patients without access to the necessary technology; VCs are inappropriate forums for sensitive consultations in oncology, etc), it indirectly benefits them because of the reduced waiting times for appointments and more frequent appointments if necessary.

VCs also have the potential to free up space on waiting lists, thereby reducing the time between appointments. It could also provide patients with easier and more frequent access to their health care providers. We speculate that this could have positive effects on compliance and communication and overall improve the doctor-patient relationship.

Patient satisfaction is a crucial part of making the VC a part of the future. The key aim of any innovation in health care technology must be to enhance ease of accessibility to the health care system and improve outcomes. Patient satisfaction is a strong predictor of improved outcomes, including compliance and treatment adherence [28,29]. To allow for a thorough assessment, a patient satisfaction component was added to the survey to provide some insight into how patients feel about different aspects of their health care.

We looked at how importantly patients rate their involvement in their health care as opposed to having their doctor assume control, and we found that patients consider it very important, with 82.2% (152/185) in strong agreement with the statement. Due to the arguably impersonal nature of the VC, there may be a reduced ability for the patient to be as involved as they would like to be. Conversely, there is a plausible case that taking control of appointment times/location can facilitate a greater sense of empowerment in health care decisions, as could be the case in VCs.

When asked how comfortable they were with sharing personal/sensitive information with their doctor, all patients agreed that they would be comfortable sharing personal/sensitive information, with 78.0% (174/223) strongly agreeing, 11.2% (25/223) moderately agreeing, and <1% disagreeing. The rest gave no answer. However, 35.0% (78/223) stated that they would be uncomfortable sharing personal information about their health in a virtual setting. Nevertheless, other authors have found that virtual settings encourage discussions about sensitive or potentially embarrassing information [30]. The identification of potential barriers to VC usage, including addressing patient fears regarding confidentiality, is key in the development of this service. Further research in this area is certainly warranted.

Another likely barrier to use is the central role of the physical examination in the doctor-patient relationship. This relationship is long recognized and well described in the literature [31]. Interestingly, in this cohort, it was found that 88.8% (198/223) of patients are indeed of the view that physical examination is important during a consultation, but 51.1% (114/223) believe that the doctor is able to perform their job even if they are not able to conduct a physical examination. Again, the concept of VCs must be applied to a carefully selected group of patients; eg, new patients with red flag symptoms clearly warrant a physical examination.

Despite the benefits of VC to patients in terms of time and expense, the data showed that in the prospective cohort, 55.2% (123/223) of patients expressed a preference for the OPD as compared with 30.9% (69/223) preferring VC. This may be partially explained by the fact that the average age of the sample population was above 50 years. This age group may be less familiar with technology and smartphones; this may explain their reluctance to make the change from the more traditional setting. Retrospectively, the fact of being an older patient was not necessarily an impediment to successfully using the VC; however, we observed anecdotally that younger patients had greater facility with the technology, which may merit further investigation.

A separate cohort of patients who had attended the VC were surveyed with a modified version of the questionnaire used for the other patients to assess their satisfaction level and their opinion on the outcome of their health care. A total of 100% (20/20) of the patients found it to be very time- and cost-effective and believe that the outcome of their care will be exactly the same as if they were to attend an outpatient appointment and meet their doctor in person. Furthermore, those who were unable to operate the technology were often assisted by family member or friends. Therefore, as mentioned previously, older patients are not always ineligible to be a part of the VC system, though they may need additional considerations and resources.

When we compared the satisfaction ratings in traditional and virtual outpatient clinics, 83.9% (187/223) of outpatients overall were satisfied with their appointment, showing that the OPD has an overall good patient satisfaction rate, which is important as it is the current standard of care. It should be noted that the retrospective analysis of patient satisfaction in VC had a much smaller sample size, and we acknowledge the potential for bias in a telephone interview as compared with an anonymous survey. Nonetheless, our results are encouraging and suggest that in an appropriately selected cohort, VCs can offer a viable alternative to the traditional model in the outpatient setting.

### Limitations

Before the findings of this study can be fully appreciated, its limitations must be acknowledged. First, the participants of this study cannot be said to be representative of all patient groups. Patients were recruited on a voluntary basis after an announcement at the outpatient reception; thus, it is not possible to quantify exactly how many patients were in the overall sample size. Patient groups excluded from completing surveys included children; patients with poor vision; and patients with limited hand mobility, literacy, etc. It is possible that if the authors had additional resources and permissions to facilitate including these patients that this may influence results (eg, if interpreters were on hand to include the viewpoints of those with poor vision or limited English language proficiency). Similarly, very few younger adults took part in the survey, given that the majority of participants in outpatients were older adults. It is plausible to speculate that this cohort may have been more receptive to the idea of VCs; this would represent a key area of future research. It should also be noted that the larger prospective cohort was heterogeneous in nature, with some patients having had inpatient stays, which may well color their attitude toward virtual care as compared with patients who had a straightforward day procedure without complication. Even within the total pool of patients available in the sample, our findings pertain only to the population in the general surgical outpatients, and we caution against generalizing these finding to other specialties without further research.

With regard to the survey itself, its structure could have been improved by predistribution validation for reliability and relevance by a panel of both patients and professionals. The “age” and “gender” questions were commonly overlooked, which compromised a key aspect of our demographic analysis. Furthermore, there were some gaps in data, which may reflect “participant fatigue” because of a lengthy survey.

In terms of the retrospective follow-up cohort who had previously attended the VC, the survey was significantly shortened to minimize additional burden to the patients, given the need to read it to participants over the phone. Initial concerns raised by stakeholders included the feasibility of the technical aspects of the software, and so, an additional question regarding the audiovisual quality of the consultation was included. Conversely, the survey did not include the section regarding travel times, time off work, etc as this was irrelevant to the cohort. The rest of the survey focused on general satisfaction and confidence regarding confidentiality. Thus, the detail of some specific questions was lost in the retrospective cohort, such as attitudes to necessity of physical examination. However, given that the patient satisfaction in general with the VC was 100%, it is reasonable to hope that the lack of physical examination did not represent an insurmountable hurdle to these patients.

In future work, we would consider a longer survey identical to that filled out in OPD, though this raises different issues regarding poor follow-up rates (postal surveys) and privacy concerns (email responses). Further work is needed to identify areas of patient concern and further refine the VC service.

Relevance of findings would have been improved had the clinical conditions of both prospective and retrospective respondents been recorded; however, these data were outside of the data protection scope permitted by this project. Patients were noted to fall within the eligibility criteria, but the individual procedures were not enumerated as the collection of patient-specific data (medical comorbidities, etc) was outside of the permissions granted for this pilot project; thus, regrettably, we were unable to include this information in this study. Again, future work should take this shortcoming into account.

In addition, costing analysis was based on salary provision of administrative and clinical staff only, with accurate information technology maintenance costs unavailable at the time of writing. As this project was a pilot of the concept of VCs within this setting, further detailed analysis of this component and others is certainly warranted. Future work in this field should follow the nonadoption, abandonment, scale-up, spread, and sustainability framework [19] to explore the challenges inherent in health care delivery change, and indeed, it is acknowledged that this project would have been improved by use of the framework.

Another limitation that was evident was the lack of awareness about VCs among the general public and medical professionals. It is hoped that ongoing work in this area will lead to the improvement of the VC service and its expansion in the hospital service for appropriate patients.

### Conclusions

In conclusion, VCs have the capacity to deliver on its expectations of reducing patient waiting times and improving patient care. However, it requires a meticulous integration into the existing system to convince patients of the advantages that it may offer. More research is required to assess which patient cohorts and departments it is most suitable for.

## Appendix

Multimedia Appendix 1 Survey regarding patient logistics (travel time/waiting time/work missed), attitudes toward the current standard of care, and attitudes toward the virtual clinic.

## Abbreviations

GP: general practitioner
MDT: multidisciplinary team
OPD: outpatient department
VC: virtual clinic

## References

[1] Branning G, Vater M (2016). Healthcare spending: plenty of blame to go around. Am Health Drug Benefits.

[2] Islam SM, Purnat TD, Phuong NT, Mwingira U, Schacht K, Fröschl G (2014). Non-communicable diseases (NCDs) in developing countries: a symposium report. Global Health.

[3] Hara K, Otsubo T, Kunisawa S, Imanaka Y (2017). Examining sufficiency and equity in the geographic distribution of physicians in Japan: a longitudinal study. BMJ Open.

[4] Liddy C, Rowan MS, Afkham A, Maranger J, Keely E (2013). Building access to specialist care through e-consultation. Open Med.

[5] Wallace P, Barber J, Clayton W, Currell R, Fleming K, Garner P, Haines A, Harrison R, Jacklin P, Jarrett C, Jayasuriya R, Lewis L, Parker S, Roberts J, Thompson S, Wainwright P (2004). Virtual outreach: a randomised controlled trial and economic evaluation of joint teleconferenced medical consultations. Health Technol Assess.

[6] Wood BR, Unruh KT, Martinez-Paz N, Annese M, Ramers CB, Harrington RD, Dhanireddy S, Kimmerly L, Scott JD, Spach DH (2016). Impact of a telehealth program that delivers remote consultation and longitudinal mentorship to community HIV providers. Open Forum Infect Dis.

[7] Le Doare K, Mackie NE, Kaye S, Bamford A, Walters S, Foster C (2015). Virtual support for paediatric HIV treatment decision making. Arch Dis Child.

[8] Venkataraman V, Donohue SJ, Biglan KM, Wicks P, Dorsey ER (2014). Virtual visits for Parkinson disease: a case series. Neurol Clin Pract.

[9] Westra I, Niessen FB (2015). Implementing real-time video consultation in plastic surgery. Aesthetic Plast Surg.

[10] Marsh JD, Bryant DM, MacDonald SJ, Naudie DD, McCalden RW, Howard JL, Bourne RB, McAuley JP (2014). Feasibility, effectiveness and costs associated with a web-based follow-up assessment following total joint arthroplasty. J Arthroplasty.

[11] Gerlach UA, Vrakas G, Holdaway L, O'Connor M, Macedo R, Reddy S, Friend PJ, Giele H, Vaidya A (2016). Skype clinics after intestinal transplantation - follow-up beyond post codes. Clin Transplant.

[12] Katz IJ, Pirabhahar S, Williamson P, Raghunath V, Brennan F, O'Sullivan A, Youssef G, Lane C, Jacobson G, Feldman P, Kelly J (2018). iConnect CKD - virtual medical consulting: a web-based chronic kidney disease, hypertension and diabetes integrated care program. Nephrology (Carlton).

[13] Good DW, Lui DF, Leonard M, Morris S, McElwain JP (2012). Skype: a tool for functional assessment in orthopaedic research. J Telemed Telecare.

[14] Stanberry B (2006). Legal and ethical aspects of telemedicine. J Telemed Telecare.

[15] Greenhalgh T, Procter R, Wherton J, Sugarhood P, Shaw S (2012). The organising vision for telehealth and telecare: discourse analysis. BMJ Open.

[16] Kruse CS, Karem P, Shifflett K, Vegi L, Ravi K, Brooks M (2018). Evaluating barriers to adopting telemedicine worldwide: a systematic review. J Telemed Telecare.

[17] Coventry L, Branley D (2018). Cybersecurity in healthcare: a narrative review of trends, threats and ways forward. Maturitas.

[18] Huff C (2014). ACP Internist.

[19] Greenhalgh T, Wherton J, Papoutsi C, Lynch J, Hughes G, A'Court C, Hinder S, Fahy N, Procter R, Shaw S (2017). Beyond adoption: a new framework for theorizing and evaluating nonadoption, abandonment, and challenges to the scale-up, spread, and sustainability of health and care technologies. J Med Internet Res.

[20] Ekeland AG, Bowes A, Flottorp S (2010). Effectiveness of telemedicine: a systematic review of reviews. Int J Med Inform.

[21] de la Torre-Díez I, López-Coronado M, Vaca C, Aguado JS, de Castro C (2015). Cost-utility and cost-effectiveness studies of telemedicine, electronic, and mobile health systems in the literature: a systematic review. Telemed J E Health.

[22] videoDoc - See a Doctor in Minutes.

[23] McNamara T (2016). Cork University Hospital.

[24] (2017). The Irish Times.

[25] (2017). The Irish Times.

[26] Goodbody W (2018). RTE News.

[27] Goodbody W (2019). RTE News.

[28] Ha JF, Longnecker N (2010). Doctor-patient communication: a review. Ochsner J.

[29] Kerse N, Buetow S, Mainous AG, Young G, Coster G, Arroll B (2004). Physician-patient relationship and medication compliance: a primary care investigation. Ann Fam Med.

[30] Jones G, Brennan V, Jacques R, Wood H, Dixon S, Radley S (2018). Evaluating the impact of a 'virtual clinic' on patient experience, personal and provider costs of care in urinary incontinence: A randomised controlled trial. PLoS One.

[31] Iida J, Nishigori H (2016). Physical Examination and the Physician-patient Relationship: A Literature Review. MedEdPublish.

