# Supplementary material for: Potential Benefits and Drawbacks of Virtual Clinics in General Surgery: Pilot Cross-Sectional Questionnaire Study
Source: JMIR Perioper Med. 2020 Jan 13;3(1):e12491. doi: 10.2196/12491 (PMC7728406; doi:10.2196/12491)
Supplement: Multimedia Appendix 1 [file periop_v3i1e12491_app1.docx]

**APPENDIX 1- Survey**

**AGE:**

**MRN:**

**GENDER:**

**Video clinic patients**

Age of the patient:

1. Have you ever been to the outpatient department at Beaumont Hospital : **YES NO**

2. Estimated one-way travel time for your appointment :

3. Estimated total travel costs for your appointment:

4. How many days were you away from work if any?

5. What type of absence was taken for time away (circle most appropriate response)?

Paid time off (vacation time) Sick day Personal day Time off without pay

Other:__________

6. Did you or anyone accompanying you have to miss an activity or routine responsibility other than work to attend the appointment: **YES NO**

7. How long would you estimate you waited after checking in with the receptionist?

**Patient satisfaction**

For the following questions, please circle the number that represents how you feel about the specific aspects of your medical care

8. Taking an active role in my own health care is important

Strongly disagree 1 2 3 4 5 6 7 Strongly agree

9. I was pleased with the quality of the medical encounter

Strongly disagree 1 2 3 4 5 6 7 Strongly agree

10. I was pleased with the video/ audio quality

Strongly disagree 1 2 3 4 5 6 7 Strongly agree

11. My visit today was on-time and efficient

Strongly disagree 1 2 3 4 5 6 7 Strongly agree

12. I believe that the medical encounter was conducted in a confidential manner

Strongly disagree 1 2 3 4 5 6 7 Strongly agree

13. I was able to share sensitive and/or personal information with my provider

Strongly disagree 1 2 3 4 5 6 7 Strongly agree

14. I was overall satisfied with appointment today

Strongly disagree 1 2 3 4 5 6 7 Strongly agree

15. I believe the provider is able to do his or her job even if they are not able to conduct a physical examination

Strongly disagree 1 2 3 4 5 6 7 Strongly agree

16. I believe that the outcome of the virtual clinic is the same as that of a standard outpatient clinic

Strongly disagree 1 2 3 4 5 6 7 Strongly agree

17. Considering the cost and time commitment of my appointment today, I would choose to meet with my provider in this setting in the future (Virtual Clinic)

Strongly disagree 1 2 3 4 5 6 7 Strongly agree
